# Lung Ventilation/Perfusion Scintigraphy for the Screening of Chronic Thromboembolic Pulmonary Hypertension (CTEPH): Which Criteria to Use?

**DOI:** 10.3389/fmed.2022.851935

**Published:** 2022-03-07

**Authors:** Romain Le Pennec, Cécile Tromeur, Charles Orione, Philippe Robin, Raphaël Le Mao, Claire De Moreuil, Mitja Jevnikar, Clément Hoffman, Laurent Savale, Francis Couturaud, Olivier Sitbon, David Montani, Xavier Jaïs, Grégoire Le Gal, Pierre Yves Salaün, Marc Humbert, Pierre Yves Le Roux

**Affiliations:** ^1^Service de médecine nucléaire, EA3878 (GETBO) IFR 148, CHRU de Brest, Université de Bretagne Occidentale, Brest, France; ^2^Département de Médecine Interne et Pneumologie, EA 3878 (GETBO), CHRU de Brest, Université de Bretagne Occidentale, Brest, France; ^3^AP-HP, Service de Pneumologie, DHU Thorax Innovation, Hôpital Bicêtre, INSERM U999, LabEx LERMIT, Centre Chirurgical Marie Lannelongue, Université Paris-Sud, Paris, France; ^4^Centre d'Investigation Clinique, Centre Hospitalier Régional et Universitaire de Brest, Brest, France; ^5^Department of Medicine, Ottawa Hospital Research Institute, University of Ottawa, Ottawa, ON, Canada

**Keywords:** chronic thromboembolic pulmonary hypertension, ventilation/perfusion scintigraphy, interpretation criteria, CTEPH, planar V/Q scintigraphy

## Abstract

**Objective:**

The diagnosis of chronic thromboembolic pulmonary hypertension (CTEPH) is a major challenge as it is a curable cause of pulmonary hypertension (PH). Ventilation/Perfusion (V/Q) lung scintigraphy is the imaging modality of choice for the screening of CTEPH. However, there is no consensus on the criteria to use for interpretation. The aim of this study was to assess the accuracy of various interpretation criteria of planar V/Q scintigraphy for the screening of CTEPH in patients with PH.

**Methods:**

The eligible study population consisted of consecutive patients with newly diagnosed PH in the Brest University Hospital, France. Final diagnosis (CTEPH or non-CTEPH) was established in a referential center on the management of PH, based on the ESC/ERS guidelines and a minimum follow-up of 3 years. A retrospective central review of planar V/Q scintigraphy was performed by three nuclear physicians blinded to clinical findings and to final diagnosis. The number, extent (sub-segmental or segmental) and type (matched or mismatched) of perfusion defects were reported. Sensitivity and specificity were evaluated for various criteria based on the number of mismatched perfusion defects and the number of perfusion defects (regardless of ventilation). Receiver operating characteristic (ROC) curves were generated and areas under the curve (AUC) were calculated for both.

**Results:**

A total of 226 patients with newly diagnosed PH were analyzed. Fifty six (24.8%) were diagnosed with CTEPH while 170 patients (75.2%) were diagnosed with non-CTEPH. The optimal threshold was 2.5 segmental mismatched perfusion defects, providing a sensitivity of 100 % (95% CI 93.6–100%) and a specificity of 94.7% (95%CI 90.3–97.2%). Lower diagnostic cut-offs of mismatched perfusion defects provided similar sensitivity but lower specificity. Ninety five percent of patients with CTEPH had more than 4 segmental mismatched defects. An interpretation only based on perfusion provided similar sensitivity but a specificity of 81.8% (95%CI 75.3–86.9%).

**Conclusion:**

Our study confirmed the high diagnostic performance of planar V/Q scintigraphy for the screening of CTEPH in patients with PH. The optimal diagnostic cut-off for interpretation was 2.5 segmental mismatched perfusion defects. An interpretation only based on perfusion defects provided similar sensitivity but lower specificity.

## Introduction

Chronic Thromboembolic Pulmonary Hypertension (CTEPH) is a rare complication of acute pulmonary embolism (PE) leading to severe right ventricular failure and death in the absence of treatment (1). CTEPH is characterized by the presence of macroscopic thromboembolic lesions in the proximal or distal pulmonary arteries and microscopic pulmonary vasculopathy, which obstruct blood flow and increases pressure in the pulmonary arteries (2). The incidence of CTEPH is probably underestimated because of non-specific symptoms and a high proportion of cases with no documented history of PE (3, 4). Diagnosing CTEPH is a major diagnostic challenge. Without treatment, the estimated 5-years survival of patients with CTEPH is poor, around 30% in patients with a mean Pulmonary Artery Pressure (mPAP) >40 mmHg (5, 6). However, in contrast with other groups of PH, CTEPH is potentially curable thanks to various treatment modalities including surgery, balloon pulmonary angioplasty and medical therapy (7–10).

According to the European Society of Cardiology (ESC) and the European Respiratory Society (ERS) guidelines for the diagnosis and treatment of pulmonary hypertension, Ventilation/Perfusion (V/Q) lung scintigraphy is the imaging modality of choice to exclude CTEPH at an early stage of the algorithm for diagnosing PH (10, 11). Indeed, V/Q lung scintigraphy is superior to Computed Tomography Pulmonary Angiography (CTPA), especially with a higher sensitivity (12).

While V/Q imaging has a key role in the screening of CTEPH (13), there is no consensus on the interpretation criteria to be used. According to ESC/ERS recommendations (10, 11), V/Q lung scintigraphy is considered positive for CTEPH if there are mismatched perfusion defects, but with no indication about the size and number of defects. Tunariu et al. demonstrated the superiority of planar V/Q lung scintigraphy over CTPA using the PIOPED criteria for V/Q scan interpretation (12). In this study, a high probability scan (i.e. at least two segmental mismatched perfusion defects) was suggestive of CTEPH while results were unclear for patients with an intermediate probability scintigraphy. In a recent study, Wang et al. used a lower threshold (14). V/Q lung scintigraphy was interpreted as positive for CTEPH if there was at least one segmental or two sub-segmental mismatched perfusion defects, as proposed by the European Association of Nuclear Medicine (EANM) guidelines for the diagnosis of acute PE (14–16). However, the pulmonary artery obstruction in patients with CTEPH is typically diffuse and multi-segmental and a low burden of pulmonary vascular obstruction, e.g., one segmental defect, is very unlikely to cause PH (14). On the other hand, given that V/Q lung scintigraphy is positioned as a screening tool in the diagnosis of CTEPH, a high sensitivity should remain the priority. Furthermore, an imaging technique using a perfusion-only scan along with a low-dose CT acquisitions (Q-LDCT), has been reported to exhibit adequate performance for CTEPH screening compared to V/Q lung scintigraphy, which may question the diagnostic value of V/Q mismatched defects as compared with perfusion defect regardless of the ventilation (17). So far, no study has evaluated and compared the diagnostic performances of V/Q scintigraphy according to interpretation criteria.

The aim of this study was to assess the accuracy of various interpretation criteria of planar V/Q lung scintigraphy for screening of CTEPH in patients with PH.

## Materials and Methods

### Population

The eligible study population consisted of consecutive patients with newly diagnosed PH referred to Brest University Hospital, France for initial assessment, and included in a French National PH registry (authorization number 842063). All patients provided written informed consent.

The diagnosis of precapillary PH was established according to the 2015 guidelines [mPAP ≥ 25 mmHg and pulmonary artery wedge pressure (PAWP) ≤ 15 mmHg measured by right heart catheterization (RHC)] (18). Patients were managed according to ESC/ERS guidelines for the diagnosis and treatment of CTEPH (11), and classified into the different groups of PH based on clinical and imaging data. All patients with a possible CTEPH after initial assessment were referred to the National reference center in Paris Kremlin-Bicêtre, France, for diagnostic confirmation and to assess operability. The diagnosis of CTEPH was confirmed according to ESC/ERS guidelines (11). All patients diagnosed with CTEPH had pre-capillary PH diagnosed with RHC and typical morphological lesions of CTEPH on high resolution CT and/or conventional pulmonary angiography. All patients were followed up for minimum 3 years with multiple check-up review and RHC to assess evolution and avoid misdiagnosis.

Demographic data and history of acute PE were collected from the French PH registry. Hemodynamics results from RHC at initial screening (pulmonary vascular resistances (PVR) expressed in dyn.sec.cm^−5^ and mPAP expressed in mmHg) were also collected in order to evaluate the correlation between the extent of perfusion defects and the alteration of hemodynamics parameters.

### V/Q Scans Acquisition and Interpretation

Planar V/Q lung scans were performed according to the SFMN guidelines on lung scintigraphy protocols (15, 19). Perfusion images were obtained after administration of 140 MBq of ^99m^Tc-macroaggregated albumin. Ventilation images were acquired either after inhalation of ^99m^Tc-Technegas or ^81m^Kr-Krypton gas. Imaging acquisition was performed in six views (anterior, posterior, left and right lateral, left and right posterior oblique).

A retrospective central review of all planar V/Q lung scintigraphy was performed by three nuclear physicians with different level of expertise, blinded to clinical results and to final diagnosis. Interpretation was determined via consensus reading. For each planar V/Q lung scintigraphy, the number, extent (sub-segmental or segmental) and type (matched or mismatched with ventilation images) of perfusion defects were reported. The extent of each defect was assessed visually. A defect was defined as segmental if it involved more than 75% of a segment and sub-segmental if it involved <75% (20).

### Data Analysis

Continuous data were expressed as mean ± standard deviation (SD), and categorical data were expressed as frequency and percentage (%). Differences between the two groups were analyzed for significance with the unpaired Student *t* test for continuous variables and with the Chi2 test for categorical variables.

For each planar V/Q lung scintigraphy, the number of segmental perfusion defects or equivalent (2 sub-segments = 1 segment) was computed. This was performed for mismatched perfusion defects, and for perfusion defects regardless of the ventilation (i.e., mismatched or matched defects). Receiver operating characteristic (ROC) curves were generated and areas under the curve (AUC) were calculated. For determination of the optimal diagnostic cut-off for interpretation, the main criterion was to select a high sensitivity cutoff, as V/Q lung scintigraphy is positioned as a screening tool in the diagnostic algorithm for CTEPH. Then, if various thresholds provided similar sensitivity, the threshold with the highest specificity was chosen. Correlation between the extent of perfusion defects and PAPm/PVR alteration was analyzed using Pearson correlation test.

## Results

### Population

A total of 288 patients referred to the Brest University Hospital were enrolled in the French National PH registry between January 2004 and January 2019. Among those 288 patients, 62 were excluded from the present study for the following reasons: 5 patients had a well-established diagnosis of a PH attributable to left heart disease with a post-capillary PH on RHC; 19 had V/Q SPECT imaging; three had a perfusion-only scan; images were not available in 28 patients; and seven patients died before undergoing assessment.

A total of 226 patients with newly diagnosed PH, who underwent V/Q planar scintigraphy for the screening of CTEPH, were therefore analyzed. Out of them, 56 (25%) were diagnosed with CTEPH at the reference center in Paris. Among 170 patients (75%) diagnosed with non-CTEPH, 92 were classified in group 1 of PH classification (41%), 24 in group 2 (10%), 40 in group 3 (18%), 4 in group 5 (2%), and 10 were classified as having mixed causes PH (mix from group 1, 2 and 3) (4%). Patients' characteristics in CTEPH and non-CTEPH groups are presented in Table 1.

**Table 1:** Patient baseline characteristics.

	**CTEPH patients (*N* = 56)**	**Non-CTEPH patients (*N* = 170)**	***p*-value**
Age (years)	68 (SD 57–81)	63 (SD 53–78)	*p* = 0.043
No PE history (%)	29 (39%)	160 (95%)	*p* <0.001
mPAP (mmHg)	41.6 (SD 31.4–51.8)	43.2 (SD 32.4–54.1)	*p* = 0.322
PVR (dyn.sec.cm^−5^)	594.2 (SD 274.2–914.2)	595.6 (SD 253.2–937.9)	*p* = 0.842
Segmental mismatched perfusion defects	6.4 (SD 4.5–8.2)	0.3 (SD −0.95–1.6)	*p* <0.001
Segmental perfusion defects	6.6 (SD 4.7–8.4)	1.1 (SD −0.6–1.2)	*p* <0.001

### Patients With CTEPH Diagnosis

**Figure 1:**
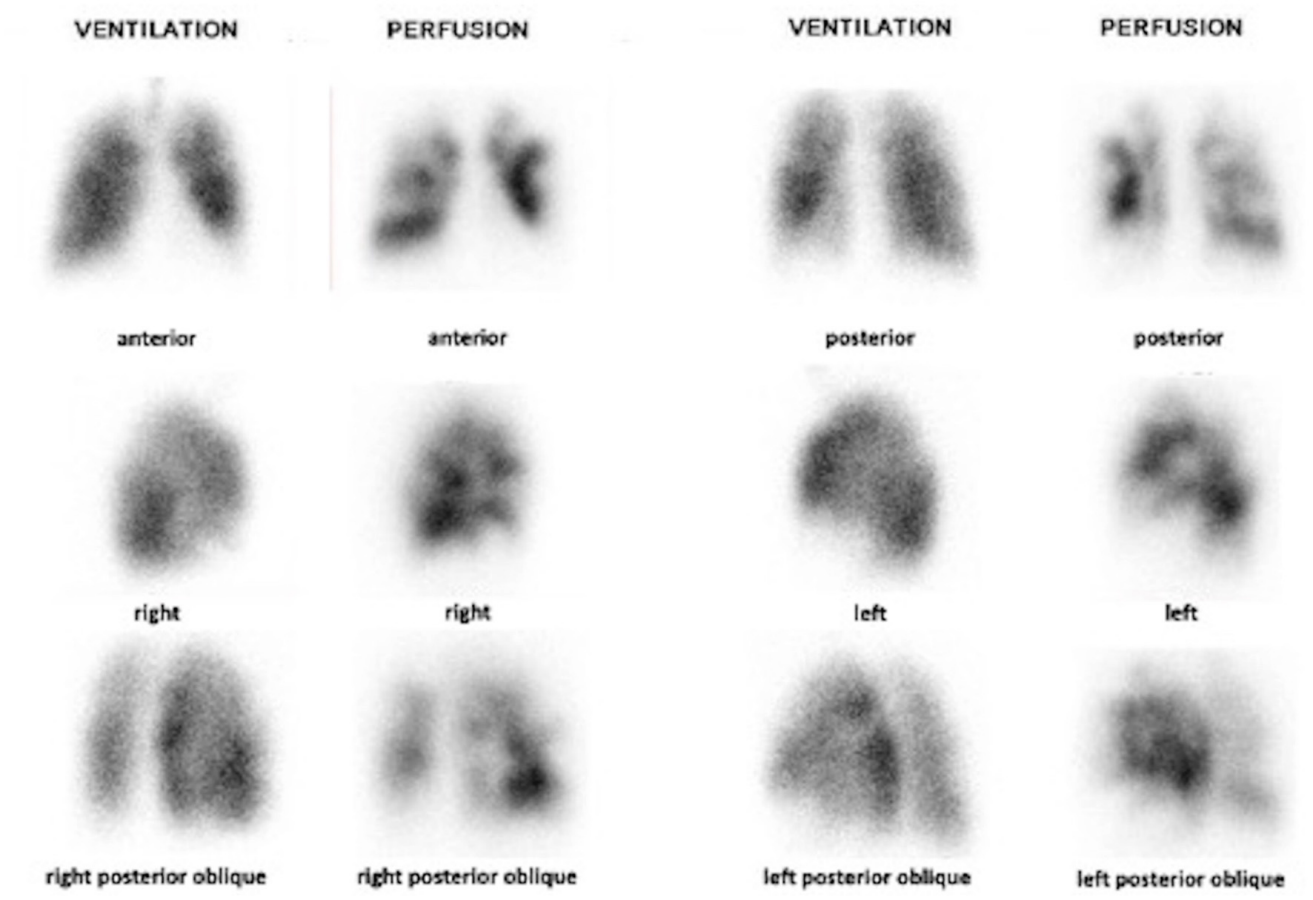
Planar V/Q scintigraphy showing multiple segmental mismatched perfusion defects in a patient with confirmed CTEPH.

Mean age of patients was 68 years old [SD (57–81)]. Mean time between first symptoms and diagnosis was 15 months. Among the 56 patients, 29 patients (39%) had no PE history. Planar V/Q lung scintigraphy was reported with a mean number of mismatched perfusion defects of 6.4 segments [SD (4.5–8.2)] and a mean number of perfusion defects of 6.6 segments [SD (4.7–8.4)]. Figure 1 illustrates a typical planar V/Q lung scintigraphy in a patient with CTEPH. Mean PAPm and PVR were 41.6 mmHg [SD (31.4–51.8)] and 594.2 dyn.sec.cm-5 [SD (274.2–914.2)], respectively. No correlation was found between the extent of perfusion defects and the degree of PAPm or PVR alteration: based on mismatched perfusion defects, correlation coefficients were 0.03 and 0.20 for PAPm and PVR, respectively.

### Patients With Non-CTEPH Diagnosis

Mean age of patients was 63 years old [SD (53–78)]. Mean time between first symptoms and diagnosis was 15 months. Among the 170 patients, 160 patients (95%) had no PE history. Planar V/Q lung scintigraphy was reported with a mean number of mismatched perfusion defects of 0.3 segments [SD (−0.95-1.6)] and a mean number of total perfusion defects of 1.1 segments [SD (−0.6–3.2)]. Mean PAPm and PVR were 43.2 mmHg [SD (32.4–54.1)] and 595.6 dyn.sec.cm^−5^ [SD (253.2–937.9)] respectively. Among the 170 non-CTEPH patients, 103 patients had a normal planar V/Q lung scintigraphy with no perfusion defect (mismatched or matched). Planar V/Q lung scintigraphy was normal in 64/92 patients (70%) from group 1, 12/24 patients (50%) from group 2, 21/40 patients (53%) from group 3, 3/4 patients (75%) from group 5, and 3/10 patients (30%) with a mixed cause of PH. Significant differences were found between CTEPH and non-CTEPH patients for PE history (*p* < 0.0001) and age (*p* = 0.043). But no significant difference was found between the two groups for PAPm (*p* = 0.322) and RVP (*p* = 0.842).

### Diagnostic Performance of Planar V/Q According to Various Criteria of Interpretation

**Figure 2:**
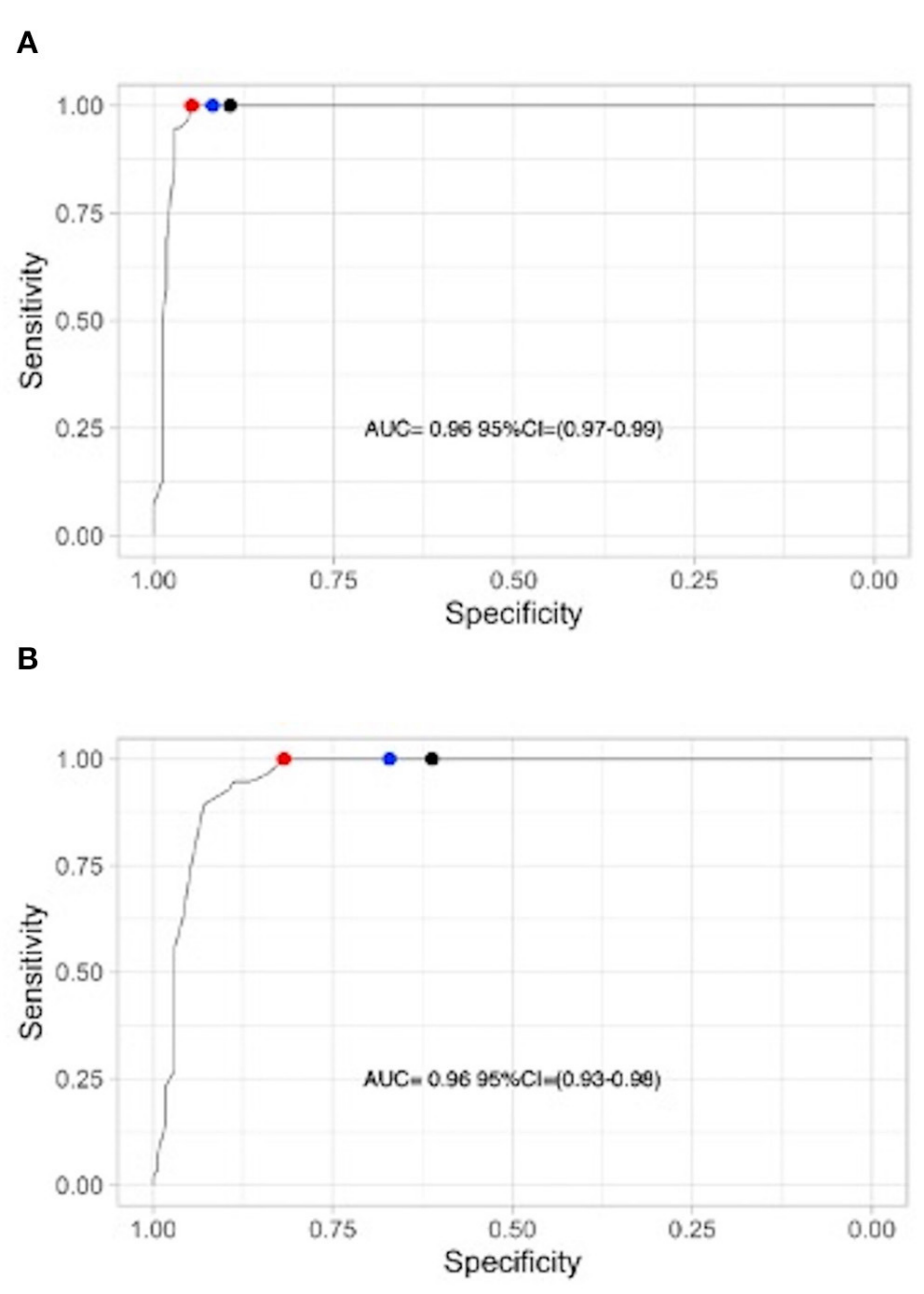
**(A)** Segmental mismatched perfusion defects. **(B)** All segmental perfusion defects (regardless of ventilation). Red points: Cut-off at 2.5 segmental; Blue points: Cut-off at 1 segmental; Black points: Cut-off at 0.5 segmental.

**Figure 3:**
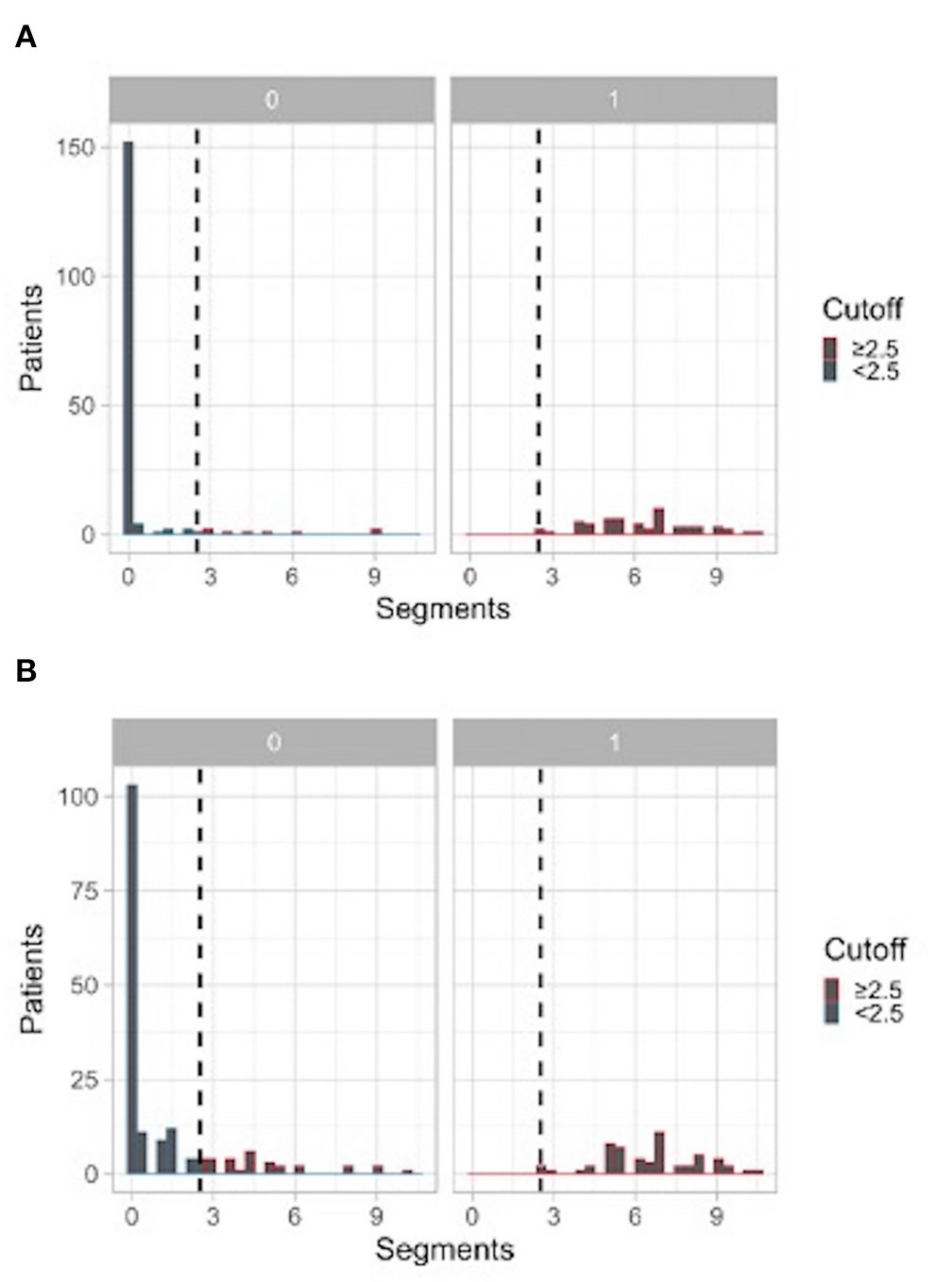
**(A)** Histogram for V/Q scan interpreted on mismatched perfusion defects. **(B)** Histogram for V/Q scan interpreted on all segmental perfusion defects (regardless of ventilation). 0 = non-CTEPH patients; 1 = CTEPH patients; Red plots = positive V/Q scan; Blue plots = negative V/Q scan.

ROC curves generated according to the number of segmental mismatched perfusion defects and segmental perfusion defects are presented in Figure 2. Figure 3 shows the histograms of distribution of mismatched segmental perfusion defects (Figure 3A) and segmental perfusion defects (Figure 3B) in the CTEPH and non-CTEPH groups. Sensitivity and specificity of lung scan according to various interpretation criteria are summarized in Table 2.

**Table 2:** Sensitivity and specificity according to criteria tested.

**Criteria**		**Sensitivity (%) CI 95%**	**Specificity (%) CI 95%**
Mismatched perfusion defects	≥ 2.5 segmental	100 (93.6–100)	94.7 (90.3– 7.2)
	≥ 1 segmental (EANM)	100 (93.6–100)	91.8 (87.7–95.0)
	≥ 0.5 segmental	100 (93.6–100)	89.4 (84.9–93.2)
Perfusion defects (regardless of ventilation)	≥ 2.5 segmental	100 (93.6–100)	81.8 (75.3–86.9)
	≥ 1 segmental (EANM)	100 (93.6–100)	66.7 (60.7–74.7)
	≥ 0.5 segmental	100 (93.6–100)	60.6 (53.1–67.6)

Based on perfusion mismatched defects, AUC was 0.98 (95%CI = 0.97–0.99). The optimal threshold was 2.5 segmental mismatched perfusion defects, providing a sensitivity of 100 % (95%CI 93.6–100%) and a specificity of 94.7% (95%CI 90.3–97.2%). Lower diagnostic cut-offs provided similar sensitivity but lower specificity: 91.8% (95%CI 87.7–95.0%) using 1 segmental mismatched defect (i.e., the EANM criteria) and 89.4% (95%CI 84.9–93.2%) using 0.5 segmental (=1 sub-segmental) mismatched defect, respectively. Out of the 56 patients with CTEPH, 53 patients (95%) had more than 4 segmental mismatched defects.

**Figure 4:**
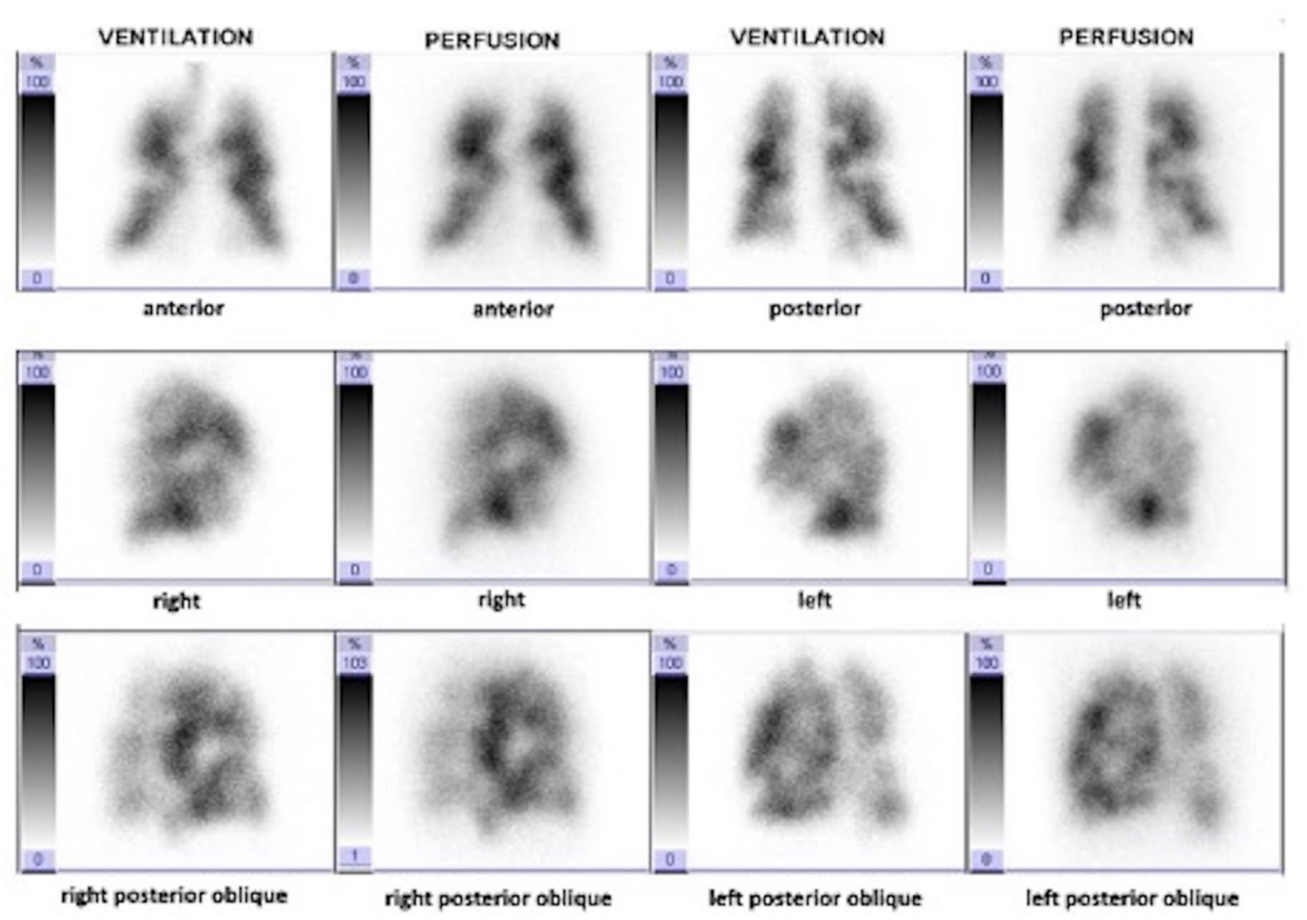
Planar V/Q scan showing multiple perfusion defects matched with ventilation impairments: final diagnosis was a PH classified as mix from group 1, 2 and 3 of PH classification, which was confirmed during the follow-up.

Based on perfusion defects regardless of ventilation, the AUC was 0.96 (95%CI 0.93–0.98). The optimal threshold was 2.5 segmental perfusion defects, providing a sensitivity of 100% (95%CI 93.6–100%) and a specificity of 81.8% (95%CI 75.3–86.9%). Lower diagnostic cut-offs provided similar sensitivity but lower specificity (See Table 2). Figure 4 illustrates a planar V/Q lung scintigraphy with multiple bilateral perfusion defects matched to the ventilation in a non-CTEPH patient.

Using the optimal positivity threshold (**≥** 2.5 segmental mismatched perfusion defects), planar V/Q lung scintigraphy was falsely interpreted as positive for CTEPH in eight patients. Among them, four patients had a final diagnosis of PH due to advanced pulmonary disease with emphysema, chronic obstructive pulmonary disease or fibrosis with both mismatched and matched defects; one patient had pulmonary veno-occlusive disease; one patient was initially diagnosed with CTEPH but was finally classified as PH from undetermined cause during the follow up; one patient had a porto-pulmonary hypertension; and one patient had pulmonary artery abnormality anatomy from congenital cause.

## Discussion

Our study confirms the high diagnostic performance of planar V/Q lung scintigraphy for screening CTEPH in patients with PH (12, 14). The optimal diagnostic cut-off for interpretation was 2.5 segmental mismatched perfusion defects, providing a sensitivity of 100% (CI 95% 93.6–100%) and a specificity of 94.7% (95%CI 90.3–97.2%), respectively. Our study also demonstrates the higher diagnostic value of mismatched perfusion defects over perfusion defects (regardless of ventilation) when screening CTEPH, as an interpretation only based on perfusion defects provided similar sensitivity but a lower specificity [81.8% (95%CI 75.3–86.9%)].

Diagnosing CTEPH is a major diagnostic challenge because it is the only curable form of PH (5, 17). Given that the V/Q lung scintigraphy is used as a screening tool for a potentially surgically curable condition, the test should be as sensitive as possible, ideally close to 100%. According to current recommendations (10, 11), all suspected cases of CTEPH on V/Q scan are then referred to an expert center to confirm the diagnosis, which implies additional testing and travels that may be invasive and costly. Therefore, V/Q lung scintigraphy should ideally also have a high specificity in order to limit unnecessary investigations.

In our study, the optimal cut-off was 2.5 segmental mismatched perfusion defects, providing a sensitivity of 100% (CI 95% 93.6–100%) and a specificity of 94.7% (95%CI 90.3–97.2%), respectively. This cut-off is roughly similar to that of a high probability planar V/Q scintigraphy according to PIOPED criteria (i.e., two segments). In the study from Tunariu et al. (12), a high probability V/Q lung scintigraphy had a sensitivity of 96.2% and a specificity of 94.6%, respectively. However, results were not straightforward for patients with an intermediate probability V/Q scintigraphy. Our study clarifies this situation, with no case of CTEPH diagnosed among patients with <2.5 segmental perfusion mismatched defects. In a recent study, Wang et al. (14) used as positivity threshold 1 segmental mismatched perfusion defect or equivalent (i.e., the EANM criteria) and reported 94.2% of sensitivity and 92.8% of specificity. Using the same criteria, we found a sensitivity of 100% (95%CI 93.6–100%) but a lower specificity of 91.8% (95%CI 87.7–95.0%). Finally, using the modified PISAPED criteria, which were also developed for the diagnosis of acute PE, the specificity was 89.4 (84.9–93.2) (21). The pulmonary artery obstruction in patients with CTEPH is typically diffuse and multi-segmental (2). In our study, the pulmonary vascular obstruction in patients diagnosed with CTEPH was 6.3 segmental mismatched perfusion defects on average (~35% of the whole lung), consistent with data from other studies (14). Furthermore, among patients with CTEPH, 95% had at least 4 segmental mismatched perfusion defects (~20% of the whole lung). Accordingly, although the V/Q lung scintigraphy is a screening tool in the management of patients with PH, not considering a single defect as a positive exam seems reasonable.

More recently, new imaging modalities such as CTPA, Magnetic Resonance Imaging (MRI) or perfusion scan with a Low-Dose Computed Tomography (Q-LDCT) have emerged as alternatives to lung scintigraphy to diagnose CTEPH (13). All these techniques rely on the analysis of lung perfusion, without information on ventilation. The need for a ventilation scan is of particular interest with the COVID-19 pandemic as the ventilation procedure increases the potential risk of contamination by the aerosol secretion and the expired air (22). According to our results, an interpretation only based on perfusion images demonstrated similar high sensitivity but lower specificity: 100% (95%CI 93.6–100) and 81.8% (95%CI 75.3–86.9%) using the same 2.5 segments cut-off. Furthermore, an interpretation based on perfusion only would have led to unnecessary refer 20 patients (12%) to the reference center. The higher specificity of mismatched perfusion defects over perfusion defects is also illustrated by the lower number of mismatched perfusion defects (0.3 segments) than of perfusion defects (1.1 segments) in the non-CTEPH group. Finally, no patient with only matched perfusion defects was diagnosed with CTEPH.

In this study, we only focused the analysis on planar V/Q lung scintigraphy and not on SPECT imaging. Based on an expert consensus, the recent ERS statement on CTEPH proposed to perform SPECT imaging and to provide retro-projected planar images from SPECT data. Indeed, SPECT has largely replaced planar lung scintigraphy in nuclear medicine facilities for the diagnostic of PE (15, 23, 24). However, the diagnostic performance of planar images generated from V/Q SPECT is controversial (25) and data for V/Q SPECT in CTEPH are still sparse. Wang et al. recently reported that both techniques were highly effective for detecting or excluding CTEPH in individual patients, with no significant differences in sensitivity or specificity (14). Although both acute PE and CTEPH are caused by the obstruction of pulmonary arteries, their underlying pathologies differ substantially (2). For instance, pulmonary artery obstructions in patients with CTEPH are more diffuse and multi-segmental as demonstrated in our study with 95% of patient with CTEPH displaying more than 4 segmental mismatched perfusion defects. For the diagnosis of acute PE, SPECT has been reported to be more sensitive and to detect more perfusion defects than planar imaging. In that respect, and given that planar V/Q lung scintigraphy is already highly sensitive, the clinical relevance of using SPECT over planar scintigraphy for screening CTEPH may be questionable. The optimal diagnostic cut-off may also be higher with SPECT than with planar imaging. On the other hand, SPECT imaging may facilitate the co-registration with other imaging modality which may be of value for pre and post-operative assessment of patients with CTEPH. SPECT imaging may also better characterize micro-vascular disease by detecting peripherical perfusion amputation (13). Finally, it would be of interest to further assess the additional value of combining a low dose CT to SPECT imaging (SPECT/CT), which may allow to better characterize morphological abnormalities for alternative diagnosis of dyspnea and therefore increase specificity (26).

Surprisingly, no correlation was found between the extent of perfusion defects and PAPm or PVR impairment in patients with CTEPH. As reported by Azarian et al. (27), it could be explained by the presence of extensive microvascular disease associated with mechanical pulmonary vascular obstruction. Indeed, in our study, 3 out of the 11 patients with PVR > 800 dyn.sec.cm^−5^ showed <6 perfusion defects. In these patients, high PVR may be explained not only by mechanical clots but also by a suspected small-vessel disease.

Our study has some limitations. Firstly, the index test and the reference standard were not completely independent as the result of the V/Q scan was used to classify patients according to the different group of PH, and especially to differentiate CTEPH and non-CTEPH cause of PH. The accuracy of V/Q lung scintigraphy could therefore have been artificially increased (28). However, our reference standard was based on the ERS/ESC guidelines for the diagnosis and treatment of PH (11). All patients with possible CTEPH were addressed to the National reference center in Paris and underwent an independent extensive work-up to assess morphology of the diseased pulmonary arteries with conventional catheter pulmonary angiography or high-resolution CT. In order to avoid misdiagnosis, all patients also had a mean follow-up of 3 years. Secondly, we performed a consensus reading and did not assess interobserver reproducibility. However, principles of interpretation, based on the recognition of mismatched perfusion defects, are similar for CTEPH screening and PE diagnosis and are therefore well-known by nuclear medicine physicians.

## Conclusion

In this study, we confirm the high diagnostic performance of planar V/Q lung scintigraphy for screening CTEPH in patients with PH. The optimal diagnostic cut-off for interpretation was 2.5 segmental mismatched perfusion defects, providing 100% sensitivity and 94.7% specificity, respectively. We also confirmed the need for a ventilation scan as an interpretation only based on perfusion defects provided lower specificity (81.8%) and would have led to unnecessary additional explorations in 12% of patients.

## Data Availability Statement

The raw data supporting the conclusions of this article will be made available by the authors, without undue reservation.

## Ethics Statement

Ethical review and approval was not required for the study on human participants in accordance with the local legislation and institutional requirements. The patients/participants provided their written informed consent to participate in this study.

## Author Contributions

Material preparation, data collection, and analysis were performed by RL, PL, PS, CO, and CT. The first draft of the manuscript was written by RL, PL, and CT. All authors contributed to the study conception, design, commented on previous versions of the manuscript, and read and approved the final manuscript.

## Conflict of Interest

The authors declare that the research was conducted in the absence of any commercial or financial relationships that could be construed as a potential conflict of interest.

## Publisher's Note

All claims expressed in this article are solely those of the authors and do not necessarily represent those of their affiliated organizations, or those of the publisher, the editors and the reviewers. Any product that may be evaluated in this article, or claim that may be made by its manufacturer, is not guaranteed or endorsed by the publisher.
